# Identification of a Novel Mutation in *TNFAIP3* in a Family With Poly-Autoimmunity

**DOI:** 10.3389/fimmu.2022.804401

**Published:** 2022-01-26

**Authors:** Marianna Nicoletta Rossi, Silvia Federici, Andrea Uva, Chiara Passarelli, Camilla Celani, Ivan Caiello, Valentina Matteo, Stefano Petrocchi, Eva Piano Mortari, Fabrizio De Benedetti, Giusi Prencipe, Antonella Insalaco

**Affiliations:** ^1^ Laboratory of Immuno-Rheumatology, Bambino Gesù Children’s Hospital, IRCCS, Roma, Italy; ^2^ Division of Rheumatology, Bambino Gesù Children’s Hospital, IRCCS, Roma, Italy; ^3^ Translational Cytogenomics Research Unit, Bambino Gesù Children’s Hospital, IRCCS, Roma, Italy; ^4^ Diagnostic Immunology Research Unit, Multimodal Medicine Research Area, Bambino Gesù Children’s Hospital, IRCCS, Roma, Italy

**Keywords:** *TNFAIP3*, poly-autoimmunity, NF-kB, IFNγ, HA20

## Abstract

Haploinsufficiency of A20 (HA20) is an inflammatory disease caused by mutations in the *TNFAIP3* gene classically presenting with Behcet’s-like disease. A20 acts as an inhibitor of inflammation through its effect on NF-kB pathway. Here we describe four consanguineous patients (three sisters and their mother) with a predominantly autoimmune phenotype, including thyroiditis, type I diabetes, hemolytic anemia and chronic polyarthritis. All patients had recurrent oral ulcers, with only 1 patient presenting also recurrent fever episodes, as a classical autoinflammatory feature. Next generation sequencing identified a novel heterozygous frameshift mutation (p.His577Alafs*95) that causes a premature stop codon in the zinc finger domain of A20, leading to a putative haploinsufficiency of the protein. Functional analyses confirmed the pathogenicity of the mutation. The variant was associated with decreased levels of A20 in blood cells. Accordingly, *ex-vivo* lipopolysaccharide (LPS)-stimulated patients’ peripheral blood mononuclear cells (PBMCs) showed higher levels of p65 NF-kB phosphorylation, as well as increased production of the proinflammatory cytokines IL-1β, IL-6 and TNF-α. Moreover, in agreement with recent observations, demonstrating a role for A20 in inhibiting STAT1 and IFNγ pathways, markedly higher circulating levels of the two IFNγ-inducible chemokines CXCL9 and CXCL10 were detected in all patients. Supporting the findings of a hyperactivation of IFNγ signaling pathway in HA20 patients, patients’ monocytes showed higher levels of STAT1 without stimulation, as well as higher phosphorylated (active) STAT1 levels following IFNγ stimulation. In conclusion, our study show that in the clinical spectrum of HA20 autoimmune features may predominate over autoinflammatory features and demonstrate, from a molecular point of view, the involvement of A20 in modulating not only the NF-kB, but also the IFNγ pathway.

## Introduction

A20 haploinsufficiency (HA20) has been described as an autosomal-dominant-inheritance autoinflammatory disease characterized by Behcet’s-like disease symptoms, such as recurrent oral and genital ulcers and inflammatory bowel disease (IBD)-like pattern (1). It is caused by loss of function mutations in the *tumour necrosis factor-a-inducible protein 3* (*TNFAIP3*) gene that encodes for the A20 protein. A20 is a highly conserved protein composed of two domains: the amino-terminal OTU (ovarian tumour) domain, with a de-ubiquitinase activity, and seven carboxyl-terminal zinc finger domains, with ubiquitin ligase and binding activities. It is involved in the negative regulation of the canonical nuclear factor-kB (NF-kB) signaling pathway, which plays fundamental roles in inflammation, immune responses and development. Since Zhou et al. first described HA20 in 2016 (1), several frameshift and nonsense mutations, in both the OTU and zinc fingers domains, have been described (2, 3).

Although HA20 has been first described as an early-onset autoinflammatory disease, recent studies described patients with late-onset presenting with autoimmune features, such as juvenile idiopathic arthritis, rheumatoid arthritis, inflammatory bowel diseases, systemic lupus erythematous, type 1 diabetes, psoriasis and coeliac disease. To date, the predominant autoimmune phenotype associated with HA20 is not clearly identifiable, as it also varies in family members carrying the same mutation (4).

In this study, we report a novel mutation in the *TNFAIP3* gene leading to a predominantly autoimmune phenotype in four subjects (three sisters and their mother) from an Italian family.

## Patients and Methods

### Patients

Patient 1 (Pt1, the index patient) is a 17 years old girl who was seen in our hospital at the age of 5, because of an autoimmune thyroiditis requiring replacement therapy. The history included recurrent oral ulcers, most often minor, since early childhood. At the age of 7 years she presented at the Emergency Room of another hospital due to worsening fatigue and shortness of breath. Hematochemistry revealed severe hemolytic anemia with positive Coombs test (5.3 g/dl) requiring blood transfusion. She was treated with intravenous (IV) immunoglobulin and subsequently with glucocorticoids because of lack of response. Three years later, she presented five relapses of hemolytic anaemia treated again with IV immunoglobulin and glucocorticoids. Because of the frequent relapses in January 2015 she was treated with rituximab. One year after the CD20 depletion treatment, asymptomatic hypogammaglobulinemia was found. Immunoglobulin replacement therapy was started, still ongoing.

When she was 9 year she developed an antinuclear-antibody (ANA) negative polyarthritis (with involvement of wrists, ankles, fingers joints and ankles tendons), treated with intra-articular glucocorticoids and methotrexate. Etanercept was added when she was 17 age for unsatisfactory disease control after a polyarticular flare affecting the two wrists, the left ankle and several tendons, with good response. After 6 months of therapy she presented an arthritis flare involving only the left ankle treated with glucocorticoid intra-articular injection and no further relapse.

Patient 2 (Pt2) is a 20 years old girl who was admitted to our hospital at the age of 9 because of Type 1 diabetes. Her medical history included, similar to her sister, recurrent oral ulcers since early childhood. In the same hospitalization, autoimmune thyroiditis was diagnosed. She was evaluated for short stature (3° centile for height at that time): a GH secretion test was normal. At the age of 19 she presented with arthritis of both wrists and tenosynovitis of ankles. Treatment with methotrexate and etanercept was started. After 9 months, she does not show signs of active arthritis.

Patient 3 (Pt3) is a 13 year-old girl. She presented oral ulcers since childhood, like her sisters, and reports recurrent febrile episodes associated with cervical lymphadenopathy, not related to infections, between the age of 6 and 8. She had an episode of pneumonia at the age 7 years that resolved with standard therapy. She was evaluated for short stature (3° centile for height); GH secretion test was normal.

Patient 4 (Pt4) is a 46 years old woman, the mother of the three girls described above. She presented recurrent oral and genital painful ulcers since childhood, along with recurrent episode of acute abdominal pain, requiring occasional evaluations at the emergency department, with no definitive interpretation. An asymptomatic autoimmune thyroiditis was diagnosed at the age of 41 following the diagnosis in two of her daughters. She started replacement therapy. Her adult height is 10-20° centile.

The study was approved by the Bambino Gesù Children’s Hospital Ethical committee. Written informed consent was obtained from the individual(s) and/or minor(s)’ legal guardian/next of kin for the publication of any potentially identifiable images or data included in this article.

### Genetics Analysis

DNA was extracted from peripheral blood with QIAgen columns (QIAsymphony DNA minikit, Qiagen, Hilden, Germany) according to the manufacturer’s instructions. Concentration and purity of DNA samples were quantified by ND-1000 spectrophotometer (NanoDrop; Thermo Scientific, Waltham, MA, USA) and by FLx800 Fluorescence Reader (BioTek, Winooski, VT, USA).

Trio-based whole-exome sequencing (WES) was performed on genomic DNA by using the Twist Human Core Exome Kit (Twist Bioscience) according to the manufacture’s protocol on a NovaSeq6000 platform (Illumina). The reads were aligned to human genome build GRCh37/UCSC hg19. The BWA Enrichment application of BaseSpace (Illumina) and the TGex software (LifeMap Sciences, Inc.) were used for the variant calling and annotating variants, respectively. Sequence data were carefully analyzed and the presence of all suspected variants was checked in the public databases (dbSNP and Genome Aggregation Database (gnomAD). Putative disease-associated sequence variants were distinguished from polymorphisms using the following filtering criteria: an allele frequency below 1% in gnomAD, species conservation of the underlying amino acid and a change in the protein’s primary structure. The variants were evaluated by VarSome (5) and categorized in accordance with the ACMG recommendations (6). Variants were examined for coverage and Qscore (minimum threshold of 30) and visualized by the Integrative Genome Viewer (IGV). The datasets presented in this study can be found in online repository (ClinVar: https://www.ncbi.nlm.nih.gov/clinvar/). The accession numbers is SUB10618687.

### PBMC Isolation

PBMCs were freshly isolated by lymphocyte separation Ficoll centrifugation and cultured in complete RPMI 1640 medium. For ELISA 5x10^5^ cells were cultured in 96 well in 200 μl complete medium. For mRNA and western blotting analyses 1x10^6^ cells were cultured in 24 well in 1 ml of complete medium.

### Western Blotting

PBMCs isolated from patients and HDs were starved for 2h in RPMI with 0,5% fetal bovine serum (FBS) and then collected for western blotting or stimulated for 30 minutes with lipopolysaccharide (LPS) 0.01μg/ml. PBMCs were then washed and lysed with RIPA buffer (Cell Signaling) and protein concentration was measured with BCA Protein assay (Pierce). 30 μg protein extracts were resolved by TGX precast mini Gels (4568084 BioRAD), transferred to nitrocellulose membranes (Amersham Life Sciences) and probed with antibody to A20 (D13H3), pS536- NF-kB (3033S), NF-kB (8242) and GAPDH (5174s), all from Cell Signaling Technology. Blots were developed with the ECL system (Amersham Biosciences) according to the manufacturer’s protocol. Band intensity were analyzed with Image LAB software.

### RNA Isolation and Quantitative Real-Time PCR

PBMCs were stimulated for 3, 6 and 12 hours with 0.01 μg/ml of LPS or for 2 hours with 10 ng/ml of Interferon-gamma (IFNγ) and mRNA expression levels of the genes of interest were analyzed by Quantitative Real-Time PCR. Total RNA was extracted using Trizol Reagent (Ambion), and cDNAs were obtained using the Superscript Vilo kit (Invitrogen). Real-time PCR assays were performed using TaqMan Universal PCR Master Mix and gene expression assays from Applied Byosystems. Gene expression was normalized using human *HPRT1* as endogenous control. Data were analyzed with the 2^Δct^ method and are expressed as arbitrary units (AU).

### Enzyme-Linked Immunosorbent Assays (ELISA)

PBMCs were stimulated for 24 hours with 0.01, 0.1 and 1 μg/ml of LPS and cytokine release in the conditioned media were analyzed by Enzyme-Linked Immunosorbent Assays (ELISAs), with commercially available kits accordingly to manufacturer instructions. Plasma from patients or healthy donors were analyzed by ELISA. IL-6 (DY206), IL-1β (DY201), TNF-α (DY210), CXCL9 (DY392) and CXCL10 (DY266) ELISA kits were all purchased from R&D Systems. The detection limits of the assays were 9,38 pg/ml (IL-6), 3,91 pg/ml (IL-1β), 15,6 pg/ml (TNF-α), 62,5 pg/ml (CXCL9) and 31,25 pg/ml (CXCL10).

### Flow Cytometry

PBMCs were treated and analyzed as previously reported (7). Briefly, PBMCs were left unstimulated or stimulated with 10 ng/ml of human recombinant IFNγ (R&D Systems) for 10 minutes at 37°C. Anti-CD3, anti-CD14 and anti-CD16 (all from Becton Dickinson) staining was performed for 20 minutes at 4°C, in order to discriminate the monocyte, neutrophil, natural killer and T cell subpopulations. Whole blood cells where then fixed with Lyse/Fix Buffer 10 min at 37°C and further incubated 10 min at RT with FcBlock 1:200 in Stain Buffer (all from Becton Dickinson). After permeabilization with Perm Buffer II (BD PhosFlow) 20 min at 4°C, samples were stained with antibodies against phosphorylated Tyrosine (701) STAT1 (pSTAT1) and total STAT1 (all from Becton Dickinson) for 20 min at 4°C. Isotype-matched control mAbs were used to determine non- specific background staining. Samples were run on a BD LSRFortessa X‐20 instrument (BD Biosciences). Results were expressed as mean fluorescence intensity (MFI) or % of positive monocytes.

### Statistical Analysis

Differences between groups were analyzed by the nonparametric Mann–Whitney U test. Significance level for statistical tests was at **p < 0.01 and ***p < 0.001 values. Graphpad Prism 9 software was used for statistical analysis and graphs.

## Results

**Figure 1:**
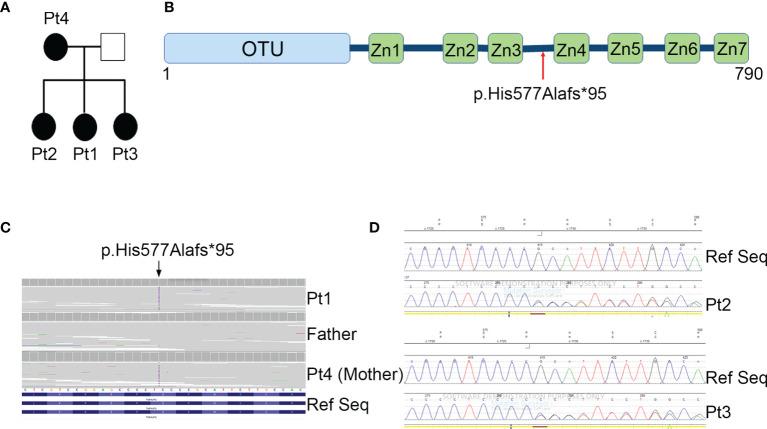
Family pedigree and genetic analyses. **(A)** Pedigree of the family with the heterozygous mutation in *TNFAIP3* gene. **(B)** Schematic diagram of A20 protein and the location of the new identified mutation. OTU, ovarian tumor domain; ZnF 1-7, zinc finger domains. **(C)** Whole exome sequencing identified a novel heterozygous mutation p.His577Alafs*95 in the *TNFAIP3* gene. **(D)** Confirmation of the presence of the mutation in Pt2 and Pt3 by Sanger Sequencing.

We studied an Italian family (**Figure 1A**) carrying a previously unreported mutation in the *TNFAIP3* gene leading to A20 haplo-insufficiency. Disease onset occurred in early childhood in four female members of the same family. Among autoimmune manifestations, thyroiditis and chronic arthritis were present in 3/4 and 2/4 patients, respectively, and diabetes and hemolytic anemia in 1 patient. All patients presented recurrent oral aphtosis, that in 1 patient were associated to genital ulcers; Pt3 was the only with a classical, albeit mild, autoinflammatory phenotype (transient recurrent episodes of fevers) and no autoimmune manifestation up to the age of 14. Patients had stunted growth with final height below or slightly above 3^rd^ percentile. Finally, Pt1 developed hypogammaglobinemia, not associated with recurrent infection, following rituximab administration (**Table 1**).

**Table 1:** Clinical and laboratory characteristics and treatment of the patients.

	Gender	Age at Onset	Autoantibody Positivity	Clinical Manifestations	Therapy
**Pt 1**	F	Early childhood (less than 2 years)		Recurrent oral ulcers	None
				Short stature (<3° centile)	None
			Ab anti-TG: 1153 U/ml Ab anti-TPO: >3000 U/ml	Autoimmune thyroiditis	L-Thyroxine
			ANA negative	Polyarthritis	Intra-articular glucocorticoid injections, methotrexate, etanercept
				Haemolitic anemia	High dose IV Immunoglobulin, high dose glucocorticoids, Rituximab
				Hypogammaglobulinemia	Substitutive IV immunoglobulin
**Pt 2**	F	Early childhood (less than 2 years)		Recurrent oral ulcers	None
				Short stature (3°-25° centile)	None
			Ab anti-TG: 1956 U/ml Ab anti-TPO: >3000 U/ml	Autoimmune thyroiditis	L-Thyroxine
			ANA negative dsDNA negative ENA negative	Oligoarthritis	Intra-articular glucocorticoid injections, methotrexate, etanercept
				Type I diabetes mellitus	Insuline
**Pt 3**	F	Early childhood (less than 2 years)		Recurrent oral ulcers	None
				Short stature (<3° centile)	None
				Recurrent fever in childhood	None
**Pt 4**	F	Early childhood (less than 2 years)		Recurrent oral and genital ulcers	None
				Short stature (10-25°)	None
			Ab anti-TG: 77 U/ml Ab anti-TPO: 1016 U/ml	Autoimmune thyroiditis	L-Thyroxine
				Recurrent episodes of abdominal pain	None

Ab anti-TG, anti-thyroglobulin antibodies (normal value <100 U/ml); Ab anti-TPO, anti-thyroperoxidase antibodies (normal value <40 U/ml); IV, intravenous.

Next generation sequencing (NGS) analysis was performed first on Pt1 and her parents. A heterozygous c.1727dupC variant in the *TNFAIP3* gene (NM_006290), introducing the frameshift variant p.His577Alafs*95, was identified in the Pt1 and her mother (Pt 4) (**Figures 1B, C**). The variant, located between the zinc finger domains 3 and 4 of the A20 protein (**Figure 1B**), was predicted to be damaging by *in silico* tools, since it introduces a premature stop codon leading to a putative haploinsufficiency of the protein. The same frameshift mutation was also present in the two sisters, Pt2 and Pt3, of our proband, as confirmed by Sanger sequencing (**Figure 1D**).

**Figure 2:**
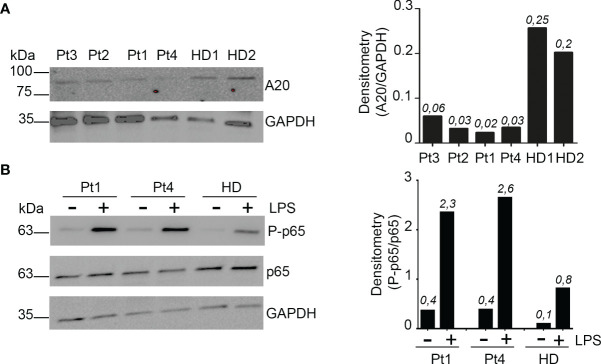
Reduced A20 function *in vivo*. **(A)** Western Blot and densitometric analysis of A20 protein levels in PBMCs isolated from patients (Pt1-4) and 2 healthy donors (HD). GAPDH was used as loading control. Densitometric quantification of A20 was reported (right graph). **(B)** PBMCs isolated from Pt1, Pt4 and one healthy subject (HD) were starved for 2 h in media with 0.5% of FBS and lysed or stimulated for 30 minutes with 0.01 μg/ml LPS and then lysed. Phosphorylated (S536) p65 NF-kB (P-p65) and total p65 NF-kB protein levels were assessed by Western blot analyses. GAPDH was used as loading control. The phosphorylated-p65 NF-kB/total p65 NF-kB densitometry comparative ratio was also reported (right graph). Similar results were obtained in two independent experiments.

Since this mutation has not been reported before, we determined its functional relevance. We first analyzed by western blot the expression levels of A20 in peripheral blood mononuclear cells (PBMCs). Compared to healthy donors, all the patients showed a strongly reduced expression of A20 in PBMCs (**Figure 2A**).

Since A20 is a known critical negative regulator of NF-kB signaling (8), we investigated if A20 decreased levels were associated with a dysregulation of NF-kB activation, by assessing the phosphorylation status of the p65 NF-kB subunit (Serine 536, S536) in unstimulated and LPS-stimulated PBMCs from Pt1 and Pt4. Higher p65 NF-kB (S536) phosphorylation levels were detected in PBMCs isolated from these patients compared to healthy donors (HD), both in unstimulated and LPS-stimulated cells (**Figure 2B**).

**Figure 3:**
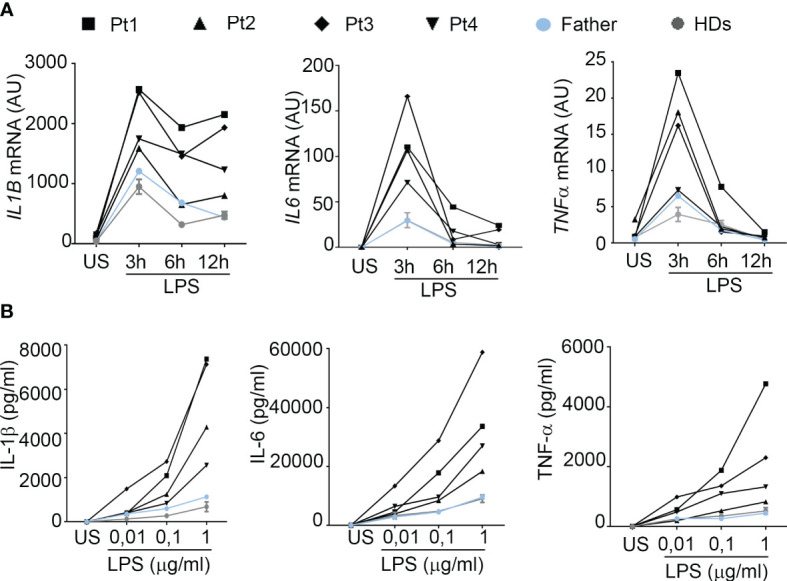
PBMCs from HA20 patients express markedly higher levels of pro-inflammatory cytokines. **(A)** The mRNA expression levels of *IL1B, IL6* and *TNF* were evaluated by qPCR analysis in PBMCs from patients (Pt1-4), a relative (father) and 2 healthy donors (HDs) unstimulated (US) or stimulated for the indicated hours h with 0.01 μg/ml of LPS. Results were obtained after normalization with the housekeeping gene *HPRT1* and were expressed as arbitrary units (AU). **(B)** PBMCs isolated from patients (Pt1-4), a relative (father) and 4 healthy donors (HDs) were unstimulated (US) or stimulated for 24 hours with 0.01, 0.1 and 1 μg/ml of LPS and IL-1β, IL-6 and TNF-α protein levels were measured in the conditioned media by ELISA.

To investigate the effects of the upregulation of NF-kB signaling in patients’ PBMCs, we analyzed the kinetics of mRNA accumulation of three cytokines known to be induced by NF-kB (9), such as IL-1β, IL-6 and TNF-α. As shown in **Figure 3A**, we observed that, compared to healthy donors, PBMCs from patients expressed markedly higher mRNA levels of all cytokines analyzed, particularly at shorter times (3 hours) following LPS stimulation. Consistent with these results, when we measured the cytokine levels released in conditioned media of PBMCs stimulated for 24 hours with increasing amount of LPS, we found that patients’ PBMCs produced markedly higher levels of IL-1β, IL-6 and TNF-α, compared to control PBMCs (**Figure 3B**). Interestingly, while in PBMCs from healthy donors there were no substantial differences in the cytokine levels produced following stimulation with increasing concentrations of LPS, in patients’ PBMCs we observed a progressive and marked increase in cytokine production, suggesting a persistent NF-kB activation, probably secondary to a defective A20-mediated NF-kB inhibition.

**Figure 4:**
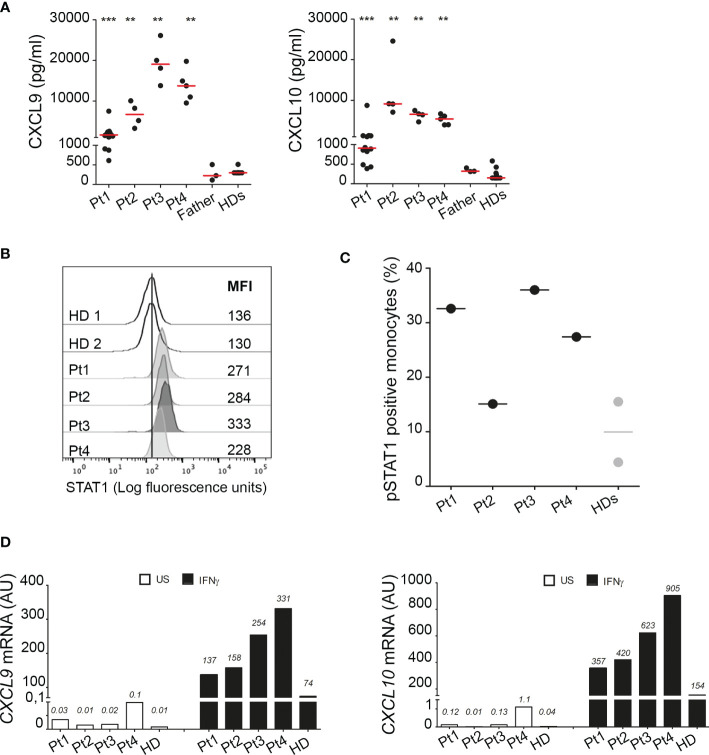
Interferon gamma pathway is upregulated in HA20 patients. **(A)** CXCL9 and CXCL10 levels were measured in plasma samples collected from patients (Pt1-4) during different hospitalizations, from the father and from healthy donors (HD; n=15) by ELISA. Red bars indicate sample median. Statistical analyses were performed with Mann-Whitney test comparing each patient with HDs. **p<0,01; ***p<0,001. **(B)** Unstimulated PBMCs from patients (Pt1-4) and two healthy donors (HDs) were stained for total STAT1 levels. Results are reported as STAT1 mean fluorescence intensity (MFI) in monocytes (CD14+ cells). **(C)** PBMCs from patients (Pt1-4) and two healthy donors (HDs) were stimulated for 10 minutes with 10 ng/ml of IFNγ and phosphorylated STAT1 (pSTAT1) levels were detected by flow cytometry. Results were reported as % of pSTAT1 positive monocytes (CD14+ cells). **(D)** PBMCs from patients (Pt1-4) and one healthy donor (HD) were left unstimulated (US, white columns) or stimulated for 2 hours with 10 ng/ml of IFNγ (IFNγ, black columns) and *CXCL9* and *CXCL10* mRNA levels were analyzed by qPCR. Results were obtained after normalization with the housekeeping gene *HPRT1* and were expressed as arbitrary units (AU). Similar results were obtained in two independent experiments.

Consistent with observations in the literature, demonstrating a role for A20 in inhibiting STAT1 and showing higher circulating levels of IFNγ inducible chemokines in patients with HA20 and in A20 genetically deficient mice (10, 11), all our patients showed markedly higher circulating levels of CXCL9 and CXCL10 compared to healthy donors (**Figure 4A**). Notably, although high levels of both CXCL9 and CXCL10 were detected in all four patients compared to healthy subjects, Pt1 showed lower levels compared to other family mutated members, possibly due to the ongoing treatments with methotrexate and hydroxylchloroquine. The other patients were not receiving any immunomodulatory treatment at time of sampling. To further investigate the activation of the IFNγ signaling pathway, PBMCs of patients were unstimulated or stimulated *ex vivo* with IFNγ and total STAT1 and phosphorylated STAT1 (Tyrosine-701) were evaluated in monocytes. We found markedly high levels of total STAT1 in unstimulated monocytes of all four patients, compared to healthy donors (**Figure 4B**). In addition, following *ex vivo* stimulation with IFNγ, the percentage of monocytes positive for phosphorylated STAT1 (pSTAT1) was marked increase in 3 out of 4 patients, compared to that observed in healthy donors (**Figure 4C**). Accordingly, in *ex vivo* experiments, PBMCs from patients showed markedly higher mRNA expression levels of *CXCL9* and *CXCL10* in unstimulated condition as well as following stimulation with IFNγ (**Figure 4D**), further supporting the hyperactivation of the IFNγ signaling pathway in HA20 patients.

## Discussion

Herein, we describe the clinical features of four family members carrying a novel mutation in the *TNFAIP3* gene leading to HA20. HA20 is an autosomal dominant genetic disease in which an insufficient production of A20 results in a decreased inhibition of the NF-kB signaling pathway. It classically presents with autoinflammatory features, particularly with a Behcet-like disease, often characterized by severe early-onset IBD. In addition to gastrointestinal manifestations, muscle-skeletal disorders with arthralgia and arthritis, ocular and skin involvement and recurrent fever are often present.

In contrast, in our family, the clinical picture is predominantly characterized by autoimmune features with the following manifestations: thyroiditis in 3 patients and chronic arthritis in 2 patients. In addition, Pt1 presented with recurrent hemolytic anemia and Pt2 with diabetes mellitus. Only Pt3 presented with recurrent episodes of fevers in childhood. It should be noted that all patients complained of oral ulcers since early childhood, and one patient presented genital ulcers, a typical features of HA20. A short stature is a common characteristic of all affected members.

The association of HA20 with autoimmunity is known in humans, as well as in animal models. Indeed, findings in mice models indicate that A20 plays a crucial role in the development and function of B cells (12). Indeed, loss of A20 in B cells leads to autoimmune pathology in old mice and defects in the generation and/or localization of the mature B cell subsets (13). In humans, the presence of autoantibodies at varying degrees has been described in patients with HA20 (14). Because of the co-occurrence of autoinflammatory and/or autoimmune features in HA20, several patients received a variety of initial diagnoses including autoimmune diseases, such as rheumatoid arthritis, juvenile idiopathic arthritis, autoimmune thyroiditis, systemic lupus erythematosus, inflammatory bowel disease (i.e. Crohn’s disease), as well as of autoinflammatory diseases including Behcet’s disease and periodic fever with aphtous stomatitis, pharyngitis and adenitis (PFAPA). Indeed, in three out of 4 of our patients anti-thyroid autoantibodies were also found and an initial diagnosis of autoimmune thyroiditis was made.

A20 negatively regulates NF-kB signaling through two mechanisms: a de-ubiquitination activity performed by the N-terminal OTU domain, and an ubiquitin ligation activity performed through its C-terminal Zn finger domains (8). Even if OTU and Zn finger domains of A20 have different biochemical functions and act on different proteins and pathways, their action converges on the inhibition of NF-kB activity. An increase of cytokines production, as well as of phosphorylation levels of NF-kB has been described in patients carrying mutations in both OTU and Zn finger domain (1, 15). Due to the great heterogeneity of clinical and prognostic presentation of HA20 (4), there has been an attempt to associate specific clinical manifestation with the positions of mutations (3). Autoimmune thyroid disorders and musculoskeletal disorders appear to be associated with mutation in the Zn finger domains and patients with Zn finger domain mutations usually present an early onset of the disease (3). However, a clear cut genotype/phenotype correlation has not been established, also due to the fact that most patients with OTU mutations described to date express truncated TNFAIP3 proteins that eliminate all the Zn finger domains.

Several studies highlighted that the spectrum of HA20 clinical manifestations can vary even among patients carrying the same mutation and/or belonging to the same family (15, 16) and that patients with the same pathogenic variant even respond differently to the same treatment regimen (4). Not surprisingly, our patients presented different clinical manifestations of variable degree of severity ranging from a mild (Pt3 and Pt4), moderate (Pt2) to a severe phenotype (Pt1). These data support the obvious hypothesis that additional genetic/environmental mechanisms may have a role in the pathogenesis of HA20.

No standard treatment has been established for the disease. Most cases seem to respond to glucocorticoids (3), but this therapy is burdened by severe side effects in the long term. The choice of treatment appears to be based on the patient’s dominant clinical phenotype. Colchicine, has been reported to be effective in some cases and reports with use of several immunosuppressants, such as methotrexate, cyclosporine-A, hydroxychloroquine and mycophenolate mofetil are present with variable response (3, 17). Because of the excessive production of inflammatory cytokines, cytokine inhibitors (anti-TNF, anti-IL-1, and anti-IL-6) are variably used as second-line treatments (3, 17). In our 2 patients with arthritis, a very satisfactory response to etanercept was observed.

The novel mutation identified in our patients (p.His577Alafs*95) causes the deletion of the A20 C-terminus, starting from Zn finger 4 domain and including Zn finger domains 4 and 7, which have been reported to be functionally relevant for A20 (18, 19). Consistent with previous observations, PBMCs isolated from our patients also showed higher levels of NF-kB phosphorylation that are associated with markedly higher production of IL-1β, IL-6 and TNF-α.

In agreement with recent evidence demonstrating a role for A20 in the inhibition of STAT1 expression in murine myeloid cells and in modulating IFNγ downstream genes (11), we found a marked increase in the circulating levels of the IFNγ-inducible chemokines CXCL9 and CXCL10 in all the patients studied, further supporting the functional relevance of this novel mutation. Consistent with data in mice, (11) we also showed that in circulating monocytes of HA20 patients the levels of total STAT1 are markedly higher than those observed in healthy subjects. Moreover, we also found that, following *ex vivo* stimulation with IFNγ, the percentage of monocytes expressing phosphorylated STAT1 is higher in patients than in healthy subjects, further demonstrating the hyperactivation of the IFNγ signaling pathway in our HA20 patients. Our results are consistent with recent data showing increased phosphorylation levels of STAT1 and STAT3 (20) and an elevation of the Type I IFN score in the whole blood of some patients with HA20 (21). Altogether, these evidence further support the role of A20 in regulating IFNs signaling pathways and provide the rationale for the therapeutic use of JAK1/2 inhibitors in HA20 patients unresponsive to the conventional treatments (20, 21).

In conclusion, we report a novel pathogenic mutation in *TNFAIP3* leading to HA20. We confirm its functional relevance and demonstrate, that HA20 leads not only to increased NF-kB activation, but also to hyperactivation of the IFNγ pathway. We confirm that a significant clinical heterogeneity exists even among patients carrying the same mutation. The spectrum of HA20 clinical manifestations is expanding and, as highlighted by our report, autoimmune features may predominate over classical autoinflammatory Behcet-like features. It is tempting to suggest that organ and non-organ specific autoimmunity in the presence of early-onset recurrent oral ulcers should elicit suspicion for HA20.

## Data Availability Statement

The original contributions presented in the study are publicly available. This data can be found here: ClinVar: SUB10618687, https://www.ncbi.nlm.nih.gov/clinvar/, SUB10618687.

## Ethics Statement

The study was approved by the Bambino Gesù Children’s Hospital Ethical committee. Written informed consent was obtained from the individual(s) and/or minor(s)’ legal guardian/next of kin for the publication of any potentially identifiable images or data included in this article. Written informed consent to participate in this study was provided by the participants’ legal guardian/next of kin.

## Author Contributions

MNR, SF, FB, GP, and AI conceived and designed the work. MNR, IC, VM, CP, SP, and EP performed the experiments. SF, AU, CC, and AI enrolled patients and collected clinical data. MNR, SF, FB, GP, and AI wrote the manuscript. All authors read and approved the final manuscript.

## Funding

This study was supported by Ricerca Corrente funding from Italian Ministry of Health to FB and GP.

## Conflict of Interest

The authors declare that the research was conducted in the absence of any commercial or financial relationships that could be construed as a potential conflict of interest.

## Publisher’s Note

All claims expressed in this article are solely those of the authors and do not necessarily represent those of their affiliated organizations, or those of the publisher, the editors and the reviewers. Any product that may be evaluated in this article, or claim that may be made by its manufacturer, is not guaranteed or endorsed by the publisher.

## References

[1] Zhou Q Wang H Schwartz DM. Loss-Of-Function Mutations in TNFAIP3 Leading to A20 Haploinsufficiency Cause an Early-Onset Autoinflammatory Disease. Nat Genet (2016) 48:67–73. doi: 10.1038/ng.3459 26642243 PMC4777523

[2] Ma A Malynn BA. A20: Linking a Complex Regulator of Ubiquitylation to Immunity and Human Disease. Nat Rev Immunol (2012) 12:774–85. doi: 10.1038/nri3313 PMC3582397 23059429

[3] Chen Y Ye Z Chen L Qin T Seidler U Tian D. Association of Clinical Phenotypes in Haploinsufficiency A20 (HA20) With Disrupted Domains of A20. Front Immunol (2020) 11:574992. doi: 10.3389/fimmu.2020.574992 33101300 PMC7546856

[4] Yu MP Xu XS Zhou Q Deuitch N Lu MP. Haploinsufficiency of A20 (HA20): Updates on the Genetics, Phenotype, Pathogenesis and Treatment. World J Pediatr (2020) 16:575–84. doi: 10.1007/s12519-019-00288-6 31587140

[5] Kopanos C Tsiolkas V Kouris A Chapple CE Albarca Aguilera M Meyer R. VarSome: The Human Genomic Variant Search Engine. Bioinformatics (2019) 35:1978–80. doi: 10.1093/bioinformatics/bty897 PMC6546127 30376034

[6] Richards S Aziz N Bale S Bick D Das S Gastier-Foster J. Standards and Guidelines for the Interpretation of Sequence Variants: A Joint Consensus Recommendation of the American College of Medical Genetics and Genomics and the Association for Molecular Pathology. Genet Med (2015) 17:405–24. doi: 10.1038/gim.2015.30 PMC4544753 25741868

[7] Pascarella A Bracaglia C Caiello I Arduini A Moneta GM Rossi MN. Monocytes From Patients With Macrophage Activation Syndrome and Secondary Hemophagocytic Lymphohistiocytosis Are Hyperresponsive to Interferon Gamma. Front Immunol (2021) 12:663329. doi: 10.3389/fimmu.2021.663329 33815425 PMC8010171

[8] Wertz IE O’rourke KM Zhou H Eby M Aravind L Seshagiri S. De-Ubiquitination and Ubiquitin Ligase Domains of A20 Downregulate NF-kappaB Signalling. Nature (2004) 430:694–9. doi: 10.1038/nature02794 15258597

[9] Liu T Zhang L Joo D Sun SC. NF-κb Signaling in Inflammation. Signal Transduct Target Ther (2017) 2:17023. doi: 10.1038/sigtrans.2017.23 PMC5661633 29158945

[10] Moll HP Lee A Minussi DC Da Silva CG Csizmadia E Bhasin M. A20 Regulates Atherogenic Interferon (IFN)-γ Signaling in Vascular Cells by Modulating Basal Ifnβ Levels. J Biol Chem (2014) 289:30912–24. doi: 10.1074/jbc.M114.591966 PMC4223299 25217635

[11] De Wilde K Martens A Lambrecht S Jacques P Drennan MB Debusschere K. A20 Inhibition of STAT1 Expression in Myeloid Cells: A Novel Endogenous Regulatory Mechanism Preventing Development of Enthesitis. Ann Rheum Dis (2017) 76:585–92. doi: 10.1136/annrheumdis-2016-209454 27551052

[12] Das T Chen Z Hendriks RW Kool M. A20/Tumor Necrosis Factor α-Induced Protein 3 in Immune Cells Controls Development of Autoinflammation and Autoimmunity: Lessons From Mouse Models. Front Immunol (2018) 9:104. doi: 10.3389/fimmu.2018.00104 29515565 PMC5826380

[13] Chu Y Vahl JC Kumar D Heger K Bertossi A Wójtowicz E. B Cells Lacking the Tumor Suppressor TNFAIP3/A20 Display Impaired Differentiation and Hyperactivation and Cause Inflammation and Autoimmunity in Aged Mice. Blood (2011) 117:2227–36. doi: 10.1182/blood-2010-09-306019 21088135

[14] Shaheen ZR Williams SJA Binstadt BA. Case Report: A Novel TNFAIP3 Mutation Causing Haploinsufficiency of A20 With a Lupus-Like Phenotype. Front Immunol (2021) 12:629457. doi: 10.3389/fimmu.2021.629457 33679772 PMC7933217

[15] He T Huang Y Luo Y Xia Y Wang L Zhang H. Haploinsufficiency of A20 Due to Novel Mutations in TNFAIP3. J Clin Immunol (2020) 40:741–51. doi: 10.1007/s10875-020-00792-9 32514655

[16] Kadowaki T Ohnishi H Kawamoto N Hori T Nishimura K Kobayashi C. Haploinsufficiency of A20 Causes Autoinflammatory and Autoimmune Disorders. J Allergy Clin Immunol (2018) 141:1485–8.e1411. doi: 10.1016/j.jaci.2017.10.039 29241730

[17] Berteau F Rouviere B Delluc A Nau A Le Berre R Sarrabay G. Autosomic Dominant Familial Behçet Disease and Haploinsufficiency A20: A Review of the Literature. Autoimmun Rev (2018) 17:809–15. doi: 10.1016/j.autrev.2018.02.012 29890348

[18] Bosanac I Wertz IE Pan B Yu C Kusam S Lam C. Ubiquitin Binding to A20 ZnF4 is Required for Modulation of NF-kappaB Signaling. Mol Cell (2010) 40:548–57. doi: 10.1016/j.molcel.2010.10.009 21095585

[19] Skaug B Chen J Du F He J Ma A Chen ZJ. Direct, Noncatalytic Mechanism of IKK Inhibition by A20. Mol Cell (2011) 44:559–71. doi: 10.1016/j.molcel.2011.09.015 PMC3237303 22099304

[20] Mulhern CM Hong Y Omoyinmi E Jacques TS D’arco F Hemingway C. Janus Kinase 1/2 Inhibition for the Treatment of Autoinflammation Associated With Heterozygous TNFAIP3 Mutation. J Allergy Clin Immunol (2019) 144:863–6.e865. doi: 10.1016/j.jaci.2019.05.026 31175876 PMC6721833

[21] Schwartz DM Blackstone SA Sampaio-Moura N Rosenzweig S Burma AM Stone D. Type I Interferon Signature Predicts Response to JAK Inhibition in Haploinsufficiency of A20. Ann Rheum Dis (2020) 79:429–31. doi: 10.1136/annrheumdis-2019-215918 PMC7507120 31767699

